# Deficits in Social Behavior Precede Cognitive Decline in Middle-Aged Mice

**DOI:** 10.3389/fnbeh.2019.00055

**Published:** 2019-03-27

**Authors:** Flora Boyer, Florence Jaouen, El Chérif Ibrahim, Eduardo Gascon

**Affiliations:** Aix Marseille Univ, CNRS, Institut de Neurosciences de la Timone (INT), Marseille, France

**Keywords:** social behavior, normal aging, cognitive impairment, neurodegeneration, mice

## Abstract

An extensive literature details deterioration of multiple brain functions, especially memory and learning, during aging in humans and in rodents. In contrast, the decline of social functions is less well understood. It is presently not clear whether age-dependent deficits observed in social behavior mainly reflect the disruption of social networks activity or are simply secondary to a more general impairment of cognitive and executive functions in older individuals. To address this issue, we carried out a battery of behavioral tasks exploring different brain functions in young (3 months) and middle-aged wild-type mice (9 months). Consistent with previous reports, our results show no obvious differences between these two groups in most of the domains investigated including learning and memory. Surprisingly, in social tasks, middle-aged animals showed significantly reduced levels of interactions when exposed to a new juvenile mouse. In the absence of overt cognitive decline, our findings suggest that social impairments may precede the disruption of other brain functions and argue for a selective vulnerability of social circuits during aging.

## Introduction

Although all organs and tissues in an organism deteriorate with age, the brain is particularly sensitive to the aging process. The anatomical organization of the brain and the unusual characteristics of neuronal physiology explain this selective vulnerability (for review see Bishop et al., 2010).

Functionally, aging has been consistently associated with impairments in different cognitive domains in humans and also in animal models (McQuail et al., 2015; Wyss-Coray, 2016; Sadoun et al., 2019). Although social activities are prominent in humans (van Schaik et al., 2012), whether these brain functions also decline with age has not been examined until recently. Impairments in certain social functions such as recognition of mental states or emotions in others (theory of mind) have been observed in aged people (for review see Moran, 2013; Fortier et al., 2016). Nonetheless, there are conflicting interpretations of these results; some studies suggest that deficits in social tasks might be, at least in part, the consequence of a more generalized age-related impairment in brain activity, namely in working memory, attention and/or executive functions (Bottiroli et al., 2016; Johansson Nolaker et al., 2018). In contrast, others seem to indicate that aging elicits a primary decline of social capabilities (Moran et al., 2012; Cavallini et al., 2013).

Similarly, behavioral studies in rodents have also shown a decrease in social interactions (Salchner et al., 2004) or social recognition memory with age (Prediger et al., 2005; Markham and Juraska, 2007). In the same line, it has been reported that older female macaques spend less time in close proximity to others and engage in fewer social interactions (Corr, 2003; Almeling et al., 2016, 2017). Together, these observations indicate that animal models can reproduce some of the age-related social cognitive deficits in humans and can help to better understand the neural correlates of such a decline.

Here, we have questioned whether aging can affect social functions in the first place. To address this problem, we compared the behavioral performance of young (3 months) and middle-aged (9 months) mice in different social and non-social tasks. We have selected this latter point over time because it has already been shown to be before the onset of cognitive decline (Lamberty and Gower, 1990; Frick et al., 1995; Bishop et al., 2010; Bizon et al., 2012). Accordingly, our results indicate that middle-aged mice have no cognitive impairments. Interestingly, animals show a significant reduction in social drive but other domains of social cognition, such as social exploration or social memory are spared. Together, these observations support the notion that certain social circuits may be particularly vulnerable to aging and that social deficits may be early symptoms of brain aging.

## Materials and Methods

### Animals

Mice were purchased from Charles Rivers Laboratories (France). A total of 48 C57Bl6/J male mice were used in this study. Half of them were young adults (*n* = 24, 10 weeks at the time of arrival) whereas the other animals were older adults (*n* = 24, 9 months). Upon arrival, each male was housed with an elderly female presenting a low fertility (>12 months) for 2 weeks. This enables acclimation to the animal facility and avoids the harmful effects of isolation while limiting reproduction and fighting. The animals were kept under specific pathogen-free conditions with a regular 12-h light/dark cycle (light on at 8:00 am) and constant conditions (21 ± 1°C; 50% humidity). Food and water were supplied *ad libitum*. Mice also benefited from some environmental enrichment (a wooden stick and a piece of cotton with each cage change).

All procedures involving mice were approved by the local ethics committee (EU0488, #6357) and are in agreement with European regulations (Directive 2010/63/EU). A special effort was made in handling animals to minimize stress or anxiety.

### Behavioral Schedule and Exclusion Criteria

All experiments were conducted between 9 am and 7 pm. Animals were habituated to the behavioral room for at least 30 min before the start of the task. The 48 animals to be analyzed were divided into three cohorts that were tested independently. For each cohort of animals, tests were conducted according to this schedule: (1) activity monitoring (Day 1); (2) three-chamber social task (Day 7); (3) intruder test (Day 9); (4) interactions in neutral arena (Day 11); (5) open field and novel object recognition (Day 13); (6) Morris water maze (MWM; Day 15–19); and (7) olfactory behavior (Day 21). We randomized the order of animals on each test.

One middle-aged animal with severe distress signs at the end of the activity monitoring was euthanized and therefore excluded from the study. Due to a software error during acquisition, it was not possible to record a video of a 3-month-old animal in the novel object recognition task. Consequently, this animal was excluded from the analysis for this task only. In the olfactory testing, trials in which an animal frequently moved the cotton were excluded from the analysis.

### Activity Monitoring

To obtain an overview of basic behavior (locomotion, feeding, drinking, resting…), mice were tracked for 20 h in an activity box (PhenoTyper, Noldus, Netherlands). Briefly, mice were placed in a dedicated cage (30 × 30 × 38 cm) containing the litter from their cage. At the top of the cage, an instrumented unit equipped with an infrared sensitive camera and three arrays of infrared LED lights enabled video recording and analysis regardless of ambient light conditions. Four PhenoTyper devices were used in parallel in this study. To avoid visual interference between animals running the test simultaneously, black curtains surrounded each PhenoTyper.

### Open Field

The open field test is a straightforward test to study locomotion and exploratory behavior in mice. The open field device consisted of a non-reflective opaque plexiglass box (40 × 40 × 40 cm) and a suspended digital camera. The animals were placed in facing an open field wall and their behavior was recorded for 10 min. The total distance covered, the time spent in the center and near the walls were calculated.

### Novel Object Recognition

For the novel object recognition task, we used the open field device and small plastic objects. In the first phase, the animals were placed facing a wall and two identical objects (green cylinders, 3.5 cm high, 4.5 cm in diameter) were placed in front of the opposite wall of the arena (10 cm from the wall and 5.5 cm apart from each other). The mice explored the arena for 10 min and were brought back to their cages for 10 min. In the second phase, one of the objects was replaced by a novel object (yellow triangular prism, 3.5 cm high, 4.5 cm on each side). The time spent exploring each object (nose point within 2 cm from the object) was quantified and used to calculate a Recognition Index (RI) as follows: RI = (time exploring object 1)/(time exploring object 1 + time exploring object 2).

### Morris Water Maze

The MWM was performed as previously described with minor modifications (LaSarge et al., 2007). Briefly, a round pool (120 cm in diameter) was filled with water (25°C) and tempera paint was added to the water until it becomes opaque. A hidden platform (10 cm in diameter) was placed about 1 cm below the water surface. The spatial cues consisted of geometric figures of different colors located in privacy blinds surrounding the water tank. The subjects were recorded by a video tracking system directly above the water tank.

During the training phase (4 days) each animal received three trials. In each trial, the animal was placed in the water tank facing the wall and allowed to explore the maze until it reached the platform. If the animal did not find the platform in 2 min, the experimenter guided it to the platform. In either case, the animal was left on the platform for 10 s. Then it was dried and returned to the cage for 5 min until the next trial. The entry point was randomized for each animal and each training day but was the same for the three trials an animal performed over a training session. Once all the animals had completed the training phase, they each performed one probe trial (2 min) on day 5, during which the platform was removed from the pool. The probe trial was performed to verify the animal’s understanding of the platform location and to observe the exploratory strategy it followed when it discovered that the platform had been removed. The latency to reach the platform and the frequency of crossings were evaluated.

### Olfactory Testing

This is a modification of the olfactory habituation/dishabituation test (Moreno et al., 2014) and consists of sequential presentations of different odors. The odor sequence used was water (reference), vanilla (non-social cue) and urine from males and females (social odors) embedded in a 2 × 2 cm piece of cotton. Each odor (or water) was presented in three consecutive trials for a duration of 2 min. The inter-trial interval was 1 min. The sniffing time of the cotton piece (nose tip less than 1 cm from the cotton) was quantified.

### Intruder Test

The protocol was adapted from previously described methods (Gascon et al., 2014). To avoid any aggressive behaviors, a juvenile CD1 male (3–5 weeks) was introduced into the home cage of the test mouse and the interactions were recorded for 5 min. The time and the number of interactive and investigative behaviors (sniffing) initiated by the target mouse were quantified.

### Interactions in Neutral Arena

In this task, the target mouse and a juvenile CD1 male (3–5 weeks) were simultaneously introduced into an open field arena (see before). The time and the number of interactive and investigative behaviors (sniffing) initiated by the target mouse during the 5 min of the test were assessed.

### Three Chamber Social Task

The three chamber apparatus is a rectangular arena (60 × 37.5 × 21 cm) made of transparent plexiglass plastic, divided into three compartments of the same size (18.5 × 37 × 21 cm). Two openings connect the center chamber with the two side chambers. In a corner of each side chamber, there is a cylindrical container (10 cm in diameter, 20 cm high). During the first trial (exploration), the target mouse was placed in the middle chamber facing a wall and allowed to explore. In the next trial (social), a stranger mouse was placed in one of the containers while the other was empty. In the last trial (memory), the same mouse was placed on one side but a novel stranger was placed in the other container. Trials lasted 10 min and animals were left for 10 min in their home cages between trials. Strangers were juvenile CD1 male mice (3–5 weeks) that had been trained to be restrained in the container. For each mouse, the location of the stranger mice in the left or right side chamber was random. For the analysis, the stranger in the social phase was arbitrarily assigned to the left chamber and the novel stranger in the social trial was placed to the right chamber. A left-right index (LRI) was calculated as the subtraction of the close exploration time (nose point within 2 cm of the container) from the left and right containers. To quantify the behavior, only the first 5 min of each trial were used.

### Device Cleaning

Between trials or animals, behavioral devices were thoroughly cleaned with water and mild soap. After rinsing, surfaces were sprayed with a 70% ethanol solution and air-dried for 5 min.

### Data Analysis and Statistics

For tests involving two freely moving animals (intruder test and interactions in the neutral arena), an investigator blind to the conditions manually scored the behaviors. For the other tests, mice tracking and behavioral analysis were carried out using dedicated software (Ethovision XT, Noldus, Netherlands). Data are presented as means ± SEM unless indicated. Statistical analyses, either nonparametric two-sample Kolmogorov-Smirnov test to compare the distribution of 3- and 9-month-old mice, or analysis of variance (ANOVA) for repeated measures followed by Sidak’s *post hoc* multiple comparison test, were performed using Prism GraphPad Software (version 7). The significance was set at *p* < 0.05 for all statistical tests.

## Results

### Middle-Aged Mice Show No Gross Deficit in Basal Activity

**Figure 1:**
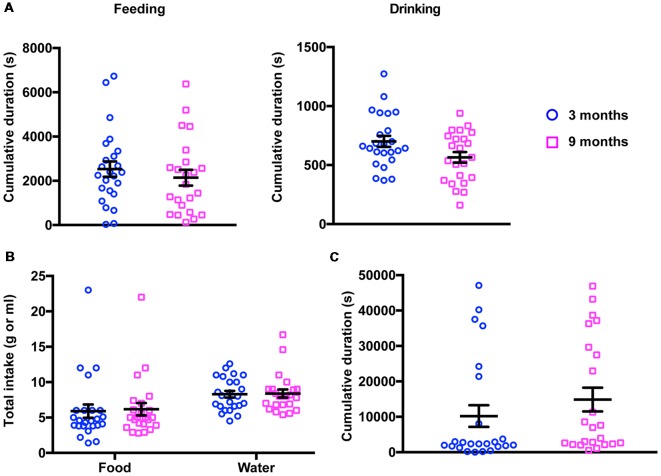
Results of 20-h activity monitoring in the PhenoTyper. **(A)** No differences were detected in the total time spent feeding (left panel) and drinking (right panel) between 3- and 9-month-old mice (3 months, feeding 2,530 ± 349.7 s, drinking 701.1 ± 46.49 s; 9 months, feeding 2,142 ± 356.0 s, drinking 565.8 ± 44.60 s). **(B)** Young and middle-aged mice showed a similar food and water consumption (3 months, food 5.91 ± 0.94 g, water 8.29 ± 0.47 ml; 9 months, food 6.18 ± 0.87 g, water 8.39 ± 0.57 ml). **(C)** The cumulative time spent in the shelter did not change in 3- vs. 9-month-old mice (3 months, 10,210 ± 3,070 s; 9 months, 14,870 ± 3,359 s).

Before submitting young (3-month-old) and middle-aged (9-month-old) mice to further behavioral phenotyping, we sought to rule out the possibility that gross behavioral abnormalities existed in these animals, either at the cohort level or at the individual level. For that purpose, we monitored both groups in their home cages over a 20-h period (PhenoTyper). We found that, during this period, there were no differences between both groups of animals in most of the parameters evaluated (Figure 1). Thus, young and middle-aged animals have a similar activity profile, including feeding, water consumption and resting in the shelter area. For these activities, neither the frequency (2-way ANOVA for repeated measures, Factor age, *F*_(1,45)_ = 0.02, *p* = 0.887) nor the time spent (2-way ANOVA for repeated measures, Factor age, *F*_(1,45)_ = 0.81, *p* = 0.372) showed significant differences between the animals at 3 and 9 months. Additionally, food and water intake were also indistinguishable between the two groups (2-way ANOVA for repeated measures, Factor age, *F*_(1,45)_ = 0.05, *p* = 0.820), suggesting that in terms of basal activity, middle-aged mice did not exhibit any impairment that would preclude the assessment of brain functions in terms of behavior.

### Middle-Aged Mice Show a Decrease in Locomotion but Normal Exploration

**Figure 2:**
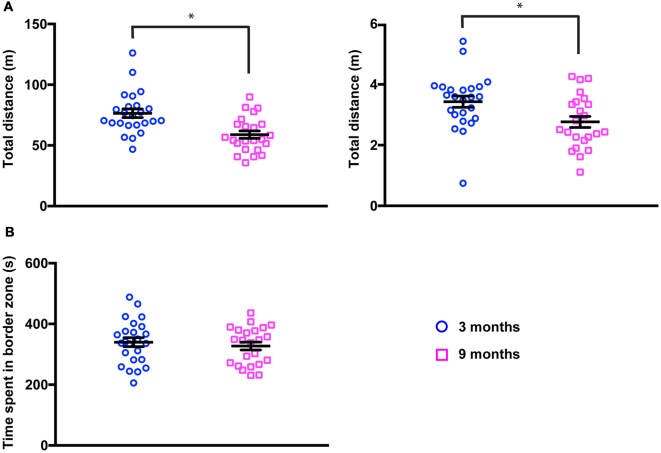
Locomotion is reduced in 9-month-old mice. **(A)** Total distance traveled in the PhenoTyper (20-h period, left panel) and in the open field (10-min period, right panel) was significantly decreased in 9-month-old mice (3 months, Phenotyper 76.48 ± 3.54 m, open field 3.43 ± 1.86 m; 9 months, PhenoTyper 58.84 ± 3.04 m, open field 2.77 ± 1.83 m). PhenoTyper *p = 0.0026, open field *p = 0.0219 (Kolmogorov-Smirnov test). **(B)** Time spent in close proximity to the walls of the open field was not modified in 3- vs. 9-month-old mice (3 months, 339.9 ± 14.82 s; 9 months, 327.35 ± 12.90 s).

It has been widely reported that locomotion decreases with age in several species. We, therefore, quantified the distance traveled by the animals during the observation period in the PhenoTyper (Figure 2A, left panel). We found that 9-month-old mice moved significantly less than younger mice (total distance, 3 months 76.48 ± 3.54 m; 9 months 58.84 ± 3.05 m, mean ± SEM, *p* = 0.0026, Kolmogorov-Smirnov test). We confirmed these findings in the open field test where animals were allowed to explore an unknown arena for only 10 min (Figure 2A, right panel). A similar reduction in the total distance traveled by animals was found in middle-aged mice (total distance, 3 months 3.43 ± 1.86 m; 9 months 2.77 ± 1.83 m, mean ± SEM, *p* = 0.0219, Kolmogorov-Smirnov test). Interestingly, the exploratory behavior of mice (the time spent near walls vs. time spent in the center of the arena) remained undistinguishable in both groups (Figure 2B) suggesting that, even if locomotion is reduced, this does not lead to a change in basic exploratory behavior.

### Middle-Aged Mice Show No Alteration in Cognitive Tasks

**Figure 3:**
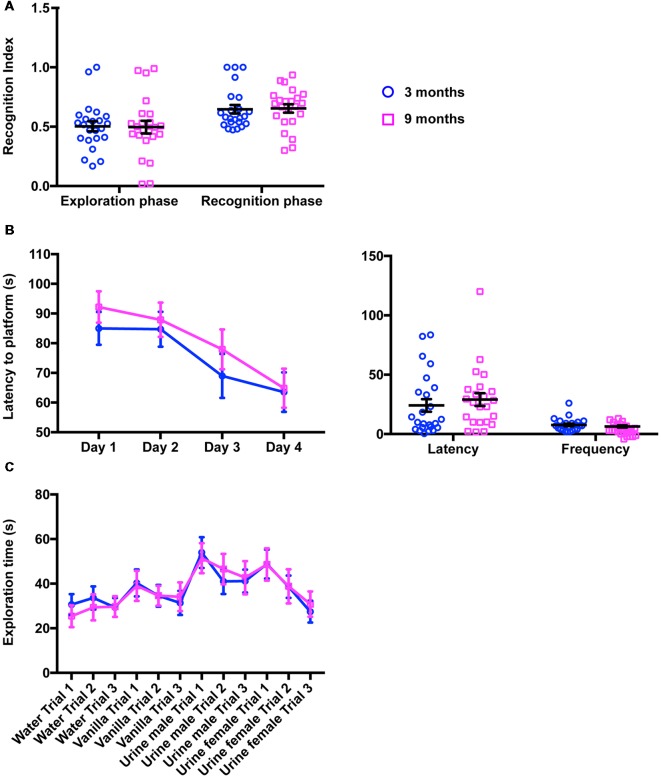
Cognitive function and olfaction are not impaired in 9-month-old mice. **(A)** In the novel object recognition task (10-min period), the proportion of time spent exploring the objects recognition index (RI) did not change in 9-month-old animals, neither during the exploration phase (two identical objects) nor during the recognition phase (one familiar object, one novel object; 3 months, 0.50 ± 0.04; RI_recognition_ = 0.65 ± 0.04; 9 months, RI_exploration_ = 0.50 ± 0.05; RI_exploration_ = 0.65 ± 0.03). **(B)** Quantification of the performances of 3- and 9-month-old mice in MWM did not reveal any difference in latency to reach the platform during training (left panel; 3 months, Day 1 85.00 ± 5.53 s, Day 2 84.70 ± 5.88 s, Day 3 68.98 ± 7.39 s, Day 4 63.51 ± 6.66 s; 9 months, Day 1 92.18 ± 5.28 s, Day 2 87.90 ± 5.77 s, Day 3 77.93 ± 6.71 s, Day 4 64.85 ± 6.57 s) or during the probe session (right panel; 3 months, Latency 24.13 ± 5.28 s, Frequency 7.71 ± 1.08; 9 months, Latency 29.06 ± 5.42 s, Frequency 6.48 ± 0.89). **(C)** In the habituation/dishabituation olfactory task, 3- and 9-month-old mice were indistinguishable and spent equivalent amount of time investigating water, vanilla and urine from males or females (3 months, Water Trial 1 30.63 ± 4.69 s, Water Trial 2 33.65 ± 5.14 s, Water Trial 3 29.34 ± 4.24 s; 3 months, Vanilla Trial 1 40.32 ± 6.02 s, Vanilla Trial 2 34.56 ± 4.85 s, Vanilla Trial 3 31.36 ± 5.37 s; Urine Male Trial 1 53.94 ± 6.89 s, Urine male Trial 2 41.00 ± 5.61 s, Urine male Trial 3 41.17 ± 5.11 s, Urine female Trial 1 48.82 ± 6.49 s, Urine female Trial 2 38.64 ± 4.98 s, Urine female Trial 3 27.46 ± 4.91 s; 9 months, Water Trial 1 25.41 ± 4.89 s, Water Trial 2 29.38 ± 5.82 s, Water Trial 3 29.73 ± 4.66 s, Vanilla Trial 1 39.03 ± 6.74 s, Vanilla Trial 2 34.61 ± 4.41 s, Vanilla Trial 3 34.12 ± 6.45 s, Urine male Trial 1 51.42 ± 6.72 s, Urine male Trial 2 46.56 ± 6.82 s, Urine male Trial 3 42.67 ± 7.42 s, Urine female Trial 1 48.60 ± 7.22 s, Urine female Trial 2 38.81 ± 7.62 s, Urine female Trial 3 30.84 ± 5.69 s).

We next examined cognitive functions using two well-established independent paradigms, the novel object recognition and the MWM. In the novel object recognition task, mice were tested for 10 min in an open field arena containing two identical (exploration phase) or different (recognition phase) objects placed in the opposite wall of the release zone. To quantify the exploratory behavior of mice, including object recognition and memory, a RI was calculated (see “Materials and Methods” section). Figure 3A summarizes the performances of 3-month-old and 9-month-old animals in this test. As expected, the young mice examined both objects similarly during the exploration phase, but showed a clear preference for the novel object during the recognition phase (RI_exploration_ = 0.50 ± 0.04; RI_recognition_ = 0.65 ± 0.04; 2-way ANOVA for repeated measures, Factor phase, *F*_(1,44)_ = 14.9, *p* = 0.0004, adjusted *p*-value for multiple comparisons: *p* = 0.0252). Middle-aged mice exhibited similar behavior (RI_exploration_ = 0.50 ± 0.05; RI_exploration_ = 0.65 ± 0.03, 2-way ANOVA for repeated measures, Factor phase, *F*_(1,44)_ = 14.9, *p* = 0.0004, adjusted *p*-value for multiple comparisons: *p* = 0.0129). No significant differences could be observed between the two groups of mice (2-way ANOVA for repeated measures, Factor age, *F*_(1,44)_ = 1.04E-4, *p* = 0.992) suggesting that moderate aging had little impact on the functions required for this task.

To further support our findings, both mouse cohorts were submitted to the MWM, a spatial memory and learning assessment task. As shown in Figure 3B (left panel), during the training phase, escape latency gradually decreased over the training days in both groups, suggesting that young and old mice learned to use spatial information to navigate to the submerged platform (2-way ANOVA, Factor training day, *F*_(3,14)_ = 6.92, *p* = 0.0002, adjusted *p*-value for multiple comparisons day 1 vs. day 4: *p*_3 months_ = 0.036 and *p*_9 months_ = 0.006). Importantly, the learning curves of young and middle-aged mice were identical (2-way ANOVA for repeated measures, Factor age, *F*_(1,45)_ = 1.22, *p* = 0.25). Similarly, in the probe trial (Figure 3B, right panel), middle-aged animals had the same latency times and platform crossovers as young mice (2-way ANOVA, Factor age, *F*_(1,90)_ = 0.231, *p* = 0.632, adjusted *p*-value for multiple comparisons: *p*_frequency_ = 0.968, *p*_latency_ = 0.600), indicating again no overt impairment in cognitive functions.

### Middle-Aged Mice Show No Impairment in Olfactory Function

Since mouse behavior largely relies on olfactory cues, we then examined olfactory functions in young and middle-aged mice. To do this, both groups of animals were exposed sequentially to water (baseline investigation), vanilla (as an attractive odorant) and urine from two different mice (as social-related cues). Our analysis revealed that both groups of animals display essentially the same olfactory exploration pattern including an increase in investigation time upon exposure to a novel odor and dishabituation in subsequent trials (Figure 3C). In addition, social cues (male and female urine) elicited much stronger responses than vanilla (Figure 3C). No difference could be found at any trial between 3-month-old and 9-month-old mice (2-way ANOVA, Factor age, *F*_(1,494)_ = 9.09E-5, *p* = 0.992) indicating that aging does not disrupt olfactory discrimination or olfactory sensitivity.

### Social Behavior Is Altered in Middle-Aged Mice

We next sought to investigate whether or not the social functions of middle-aged mice were affected. In order to obtain a comprehensive description of social behaviors, we conducted three independent paradigms, the 3-chamber task, the interactions in the neutral arena and the intruder test.

**Figure 4:**
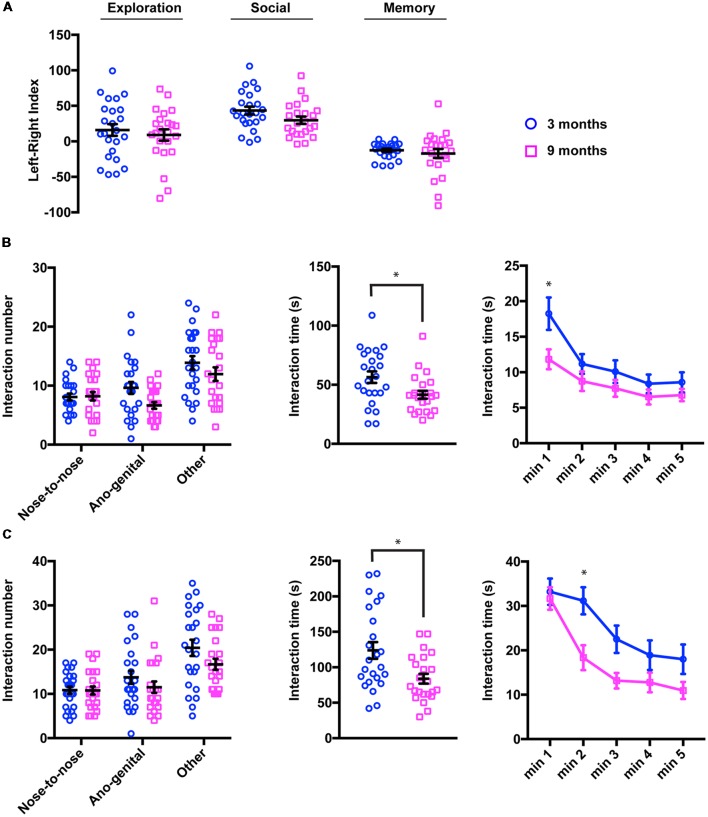
Social drive/sociability is specifically disrupted in 9-month-old mice. **(A)** In the three-chamber apparatus, the mice spent the same amount of time exploring empty objects (exploration trial) or the social partners (social and memory phases) regardless of their age. For quantification, a left-right index (LRI) was calculated (see “Materials and Methods” section; 3 months, LRI_exploration_ 16.03 ± 8.28, LRI_exploration_ 29.76 ± 5.06, LRI_social_ −12.67 ± 2.26; 9 months, LRI_social_ 9.00 ± 7.96, LRI_memory_ 43.36 ± 5.47, LRI_memory_ −17.13 ± 6.40). **(B)** Quantification of social interactions initiated by 3- and 9-month-old mice in a novel arena containing a juvenile male (3–5 week-old). Although the number and type of interactions (left panel; 3 months, Nose-to-nose 8.08 ± 0.56, Ano-genital 9.62 ± 1.02, Other, 13.87 ± 1.14; 9 months, Nose-to-nose 8.22 ± 0.72, Ano-genital 6.65 ± 0.56, Other, 11.96 ± 1.14) showed no difference between the two groups, the total time spent interacting was significantly lower in elderly mice (middle panel; 3 months, 56.46 ± 4.84 s; 9 months, 41.61 ± 3.36 s, *p = 0.0196, Kolmogorov-Smirnov test). When the interaction time was analyzed at 1-min intervals (right panel), most of the reduction was observed in the 1st min of the exploration phase (3 months, Minute 1 18.25 ± 2.28 s, Minute 2 11.16 ± 1.38 s, Minute 3 10.08 ± 1.60 s, Minute 4 8.37 ± 1.31 s, Minute 5 8.58 ± 1.40 s; 9 months, Minute 1 11.82 ± 1.39 s, Minute 2 8.74 ± 1.37 s, Minute 3 7.74 ± 1.19 s, Minute 4 6.52 ± 1.05 s, Minute 5 6.78 ± 0.86 s). *p_minute 1_ = 0.009 [2 way analysis of variance (ANOVA), Sidak test]. **(C)** In the intruder test, the number and type of interactions (left panel; 3 months, Nose-to-nose 10.83 ± 0.83, Ano-genital 13.75 ± 1.48, Other, 20.42 ± 1.82; 9 months, Nose-to-nose 10.78 ± 0.91, Ano-genital 11.52 ± 1.30, Other, 16.70 ± 1.25) were not altered in 9-month-old mice compared to 3-month-old animals. In contrast, the total time spent interacting was significantly lower in older mice (middle panel; 3 months, 123.8 ± 11.72 s; 9 months, 83.91 ± 6.84 s, *p = 0.0496, Kolmogorov-Smirnov test). At 1 min intervals (right panel), the 9-month-old mice showed no change in the exploration phase (first minute), but a significant decrease in social afterwards (3 months, Minute 1 33.20 ± 2.97 s, Minute 2 31.17 ± 3.04 s, Minute 3 22.50 ± 3.07 s, Minute 4 18.92 ± 3.43 s, Minute 5 18.00 ± 3.30 s; 9 months, Minute 1 31.70 ± 2.53 s, Minute 2 18.34 ± 2.79 s, Minute 3 13.17 ± 1.678 s, Minute 4 12.78 ± 2.22 s, Minute 5 10.96 ± 1.92 s). *p_minute 2_ = 0.006 (2-way ANOVA, Sidak test).

We first carried out the 3-chamber paradigm divided into three trials (see “Materials and Methods” section). By measuring the time spent by animals investigating empty objects or the different social partners in the side compartments (LRI, see “Materials and Methods” for the details), these different trials enabled assessment of exploratory behavior, social recognition and social memory, respectively. As shown in Figure 4A, both young and middle-aged mice displayed the expected behavioral pattern; LRI was close to 0 during the exploration phase (LRI_3 months_ = 16.03 ± 8.28; LRI_9 months_ = 9.00 ± 7.96), whereas there was a clear preference for the left chamber during the social trial (LRI_3 months_ = 43.36 ± 5.47; LRI_9 months_ = 29.76 ± 5.06) and a shift to the right side was observed in the last trial (LRI_3 months_ = −12.67 ± 2.26; LRI_9 months_ = −17.13 ± 6.40). Statistical analysis of these data confirmed that there were no significant differences at any trial between 3- and 9-month-old mice (2-way ANOVA for repeated measures, Factor age, *F*_(1,45)_ = 2.40, *p* = 0.129, adjusted *p*-value for multiple comparisons: *p*_exploration_ = 0.811, *p*_social_ = 0.329, *p*_memory_ = 0.942). Other parameters usually quantified in these tests such as the number of bouts or the time spent in each chamber were not modified either (data not shown) suggesting that fundamental social functions such as recognition of a conspecific and memory remained intact in middle-aged animals.

We next measured the social interactions of our two groups of animals in a neutral arena where they were exposed to an unfamiliar juvenile male mouse (3–5 weeks old) for 5 min. In this task, mice were confronted with a new physical and social environment and their social behavior is quantified by the time as well as the number/nature of interactions initiated by the target mouse (Figure 4B, left panel). Although young mice spent more time in social interactions than middle-aged mice (Figure 4B, central panel, 56.46 ± 4.84; vs. 41.61 ± 3.36, mean ± sem, *p* = 0.0196, two-tails Kolmogorov-Smirnov test), the number of different interactions remained unchanged (2-way ANOVA for repeated measures, Factor age, *F*_(1,45)_ = 3.84, *p* = 0.0563, adjusted *p*-value for multiple comparisons: *p*_nose_ = 0.999, *p*_ano-genital_ = 0.0613, *p*_other_ = 0.350). We further investigated this decrease in social interest by analyzing the interactions at 1 min intervals (Figure 4B, right panel). We observed that, among young animals, exploration of the social partner peaked at the 1st min and remained at lower levels thereafter. In contrast, 9-month-old mice spent much less time in social contact at the beginning of the test (2-way ANOVA for repeated measures, Factor age, *F*_(1,45)_ = 7.09, *p* = 0.0161, adjusted *p*-value for multiple comparisons: *p*_minute 1_ = 0.009, *p*_minute 2_ = 0.736, *p*_minute 3_ = 0.763, *p*_minute 4_ = 0.895, *p*_minute 5_ = 0.906). These observations suggest that social exploratory behavior, particularly social interest, is altered in middle-aged mice.

To confirm these findings, we further assessed social functions in the intruder test. In this task, a stranger male juvenile mouse (3–5 weeks-old) was introduced into the home cage, thus avoiding the need for simultaneous investigation of a novel arena. In this test, time and the number/nature of interactions were also used as an output of the social function. Figure 4C summarizes the results of this test; we found that the number of each kind of interaction was not different between young and middle-aged mice (Figure 4C, left panel, 2-way ANOVA for repeated measures, Factor age, *F*_(1,45)_ = 2.45, *p* = 0.125, adjusted *p-value* for multiple comparisons: *p*_nose_ = 0.999, *p*_ano-genital_ = 0.550, *p*_other_ = 0.136). However, consistent with our findings in the neutral arena, the total time spent in social interactions was significantly reduced (Figure 4C, middle panel, 3 months 123.8 ± 11.72 for young mice vs. 83.91 ± 6.84 for older mice, *p* = 0.0496, two-tails Kolmogorov-Smirnov test). When the interaction time was analyzed in 1-min splitting (Figure 4C, right panel), we observed that young animals explored the intruder in depth during the first 2 min of the test and that the interactions shortened subsequently. In the 9-month-old mice, social contacts were maintained at similar levels for the 1st min, but dropped significantly for the remainder of the test duration (2-way ANOVA for repeated measures, Factor age, *F*_(1,45)_ = 7.09, *p* = 0.0106, adjusted *p-value* for multiple comparisons: *p*_minute 1_ = 0.998, *p*_minute 2_ = 0.006, *p*_minute 3_ = 0.0864, *p*_minute 4_ = 0.467, *p*_minute 5_ = 0.316) arguing for an age-dependent reduction in social drive already detectable in 9-month-old mice.

## Discussion

In this study, we characterized young and middle-aged mice in social and non-social tasks. Behavioral test batteries have been used to assess a wide variety of behavioral traits. Our main findings for middle-aged mice are as follows: (i) their locomotion is significantly reduced; (ii) they have no overt impairment in multiple brain functions, including exploration, olfaction, learning and spatial and working memory; and (iii) they exhibit altered social behavior, specifically in terms of social drive/sociability.

Motor activity in the open field is known to decrease in aging mice and rats (Lalonde and Badescu, 1995; Lalonde and Strazielle, 2009). Previous work (Fahlström et al., 2011) has suggested that locomotion decline in middle-aged mice is exclusively associated with exploratory tasks, even when animals have been analyzed over longer time periods. Conversely, we found that C57Bl6/J males exhibit a reduction in the distance traveled not only in the open field (exploration-related locomotion) but also in the context of their home cage (basal locomotion). Our findings therefore argue for a gradual loss of locomotor activity with age, in accordance with an abundant literature reporting such a decline in a striking number of species, from *C. elegans* to rodents and primates (Ingram, 1988, 2000; Walton et al., 2006; Fahlström et al., 2012; Marck et al., 2017).

Many studies have addressed the impact of aging in rodents’ cognitive functions using different behavioral paradigms (Wood and Dudchenko, 2003; LaSarge et al., 2007; Bizon et al., 2012). In mice, novel object recognition and MWM have been widely used. In the former test, middle-aged mice (9–12 months) show similar performance levels to their younger counterparts (Benice et al., 2006; Davis et al., 2012; Fahlström et al., 2012). Similarly, most studies failed to detect differences in MWM until 16–18 months (Calhoun et al., 1998; Frick et al., 2002; Benice et al., 2006; de Fiebre et al., 2006; Murphy et al., 2006; Davis et al., 2012; Fahlström et al., 2012). Our findings using 9-month-old mice are consistent with these observations. More importantly, evidence suggesting that cognitive functions remain largely intact in middle-aged mice have been obtained using additional behavioral tasks (i.e., Barnes maze or radial maze; Barreto et al., 2010; Pettan-Brewer et al., 2013). Collectively, these data indicate that 9-month-old mice used in this work are tested well before the onset of any cognitive decline.

Although aging seems to spare sensory functions (Fahlström et al., 2011), decreased olfactory function is very common (>50%) in humans over 65 (Attems et al., 2015). Old mice (from 18 to 24 months) also show impairments in olfactory discrimination and memory (Enwere et al., 2004; Patel and Larson, 2009; Moreno et al., 2014). Given the relevance of olfactory cues to the social behavior of mice (Dulac and Torello, 2003; Dulac, 2006), it was essential to rule out that social alterations in middle-aged mice were not due to olfactory malfunction. We observed no differences between our two groups of mice in olfactory tasks, indicating that moderate aging may not cause olfactory deficits. Moreover, a recent report reveals that the discrimination abilities of middle-aged (10 months) mice may be even more refined than those of young (2 months) or old (23 months) animals (Rey et al., 2012), which also argues against this possibility.

The major finding in this report is the existence of social impairments in middle-aged mice. By assessing social behavior according to three independent paradigms, we were able to obtain a detailed analysis of social functions and uncover relatively subtle changes. The absence of differences in the three-chamber test suggests that middle-aged mice maintain their basic social abilities, including social exploration, recognition of a conspecific, social interest and memory. The results of the neutral arena indicate an overall decrease in the time spent on social interactions, especially during the exploratory phase (1st min of the test). A recent study reported similar results in this test in mice aged 8–12 months (Shoji et al., 2016). In this task, the mice simultaneously investigate a new environment and a social partner complicating the interpretation of the results. However, we did not observe any difference in the exploratory behavior in middle-aged mice either in the open field or in the novel object recognition (data not shown) arguing against the possibility that neutral arena deficits arise from alterations in exploratory functions. More importantly, middle-aged mice exhibited similar defects in the intruder test. Although mainly used to evaluate aggression, this test could also be used to evaluate sociability when the intruder is a non-aggressive partner (either a female or a juvenile; Moles et al., 2007; Ey et al., 2012). Here, we favor a juvenile male as a social target to avoid the confounding effect of mating in male-female dyads. We show that, even in the context of their home cage, middle-aged mice exhibit a similar reduction in interaction time compared to young animals. Interestingly, a more detailed analysis revealed that, during the 1st min of the task (active exploration of the intruder), there is no difference in the interaction time between young and middle-aged mice suggesting that social exploration and social interest are not perturbed. Then, 3-month-old animals maintain equally high levels of social interactions during the second minute whereas, in 9-month-old mice, they have dropped significantly and remain lower for the rest of the test. These findings suggest that aging may initially disrupt social drive/sociability. Remarkably, similar deficits have been described in different mouse models of fronto-temporal dementia, an early onset neurodegenerative disease primarily affecting social behavior (Filiano et al., 2013; Gascon et al., 2014; Chew et al., 2015).

It will be important in the future to narrow the window of time when social impairment begins. Nevertheless, it can be difficult to reconcile the exact same findings of an age-related decline in different mouse strains due to robust strain differences. For example, some mouse strains do not learn the spatial MWM task slowly, whereas other strains learn the task very easily, thus confounding the observation of age-related changes in performance (Upchurch and Wehner, 1988a,b). Therefore, more than defining a moment, it will be crucial investigating the brain areas involved in early decline of social activities using objective neural measurements such as functional magnetic resonance imaging and optogenetics (Benekareddy et al., 2018).

As a major limitation of our work, we have not addressed the differences between male and female in the different behavioral traits observed. Indeed, changes in behavior with age could be quantitatively different between males and females but could also vary qualitatively (in terms of signification). For example, spatial reference memory decline begins at an earlier age in females than in males, a finding that may be related to the cessation of estrous cycling (Frick et al., 2000). It has been shown that the spontaneous failure of the estrous cycle increased anxiety, suggesting that the perimenopausal period has a significant influence on anxiety-related behaviors in female (Guimarães et al., 2015). The use of factorial analyses would make it possible to identify the motivational drivers underlying the spontaneous behavior of both sexes at different periods in their lives and to identify possible relationships between the indices of the different tests. Future analyses should focus on sexual differences in behaviors expressed in conventional social tests.

Collectively, these findings open the intriguing possibility that neuronal circuits underlying specific domains of social functions (such as social drive/sociability) may be particularly susceptible to the aging process and may drive early symptoms of pathological aging.

## Data Availability

The datasets generated for this study are available on request to the corresponding author.

## Author Contributions

FB performed the behavioral tests. FB, FJ and EG analyzed the data. EI and EG wrote the manuscript.

## Conflict of Interest Statement

The authors declare that the research was conducted in the absence of any commercial or financial relationships that could be construed as a potential conflict of interest.
